# Stochastic cost-effectiveness analysis on population benefits

**DOI:** 10.1186/s12962-023-00488-y

**Published:** 2023-10-26

**Authors:** Ermo Chen

**Affiliations:** School of Mathematical Sciences, Peking University, No. 5 Yiheyuan Road, Haidian District, Beijing, 100871 People’s Republic of China

**Keywords:** Stochastic cost-effectiveness analysis, Population benefits, Risk allocation, Return on risk

## Abstract

Dealing with randomness is a crucial aspect that cost-effectiveness analysis (CEA) tools need to address, but existing stochastic CEA tools have rarely examined risk and return from the perspective of population benefits, concerning the benefits of a group of individuals but not just a typical one. This paper proposes a stochastic CEA tool that supports medical decision-making from the perspective of population benefits of risk and return, the risk-adjusted incremental cost-effectiveness ratio (ICER). The tool has a traditional form of ICER but uses the cost under a risk-adjusted expectation. Theoretically, we prove that the tool can provide medical decisions trimming that promote the risk-return level on population benefits within any intervention structure and can also serve as a criterion for the optimal intervention structure. Numerical simulations within a framework of mean–variance support the conclusions in this paper.

## Key points for decision makers

- The typical assumption in classical CEA that all get the new intervention versus standard of care may not be the best to achieve the best outcome to population, a mixed structure can be better
- The intervention structure should be modified using a criterion considering slight changes on the structure of the treatment mix.
- Use a risk adjustment concerning cost and outcome uncertainties in taking expectation in ICER calculation gives the optimal treatment mix for population benefits.

## Introduction

The classical cost-effectiveness analysis (CEA) framework is of high importance in deterministic medical decision-making, and it has been widely applied in both theoretical and practical problems. Moreover, accounting for uncertainty has gained much attention. Some tools are developed in the framework probabilistic dominance analysis, using the CEA result under different random scenarios to get a judgement with uncertainty. For example, the cost-effectiveness acceptability curve (CEAC, see Stinnett and Mullahy [1]) uses different monetary standard in judging the probability of a new intervention to be cost-effective, and finally propose the curve describing the probability of the intervention to be accepted under different monetary standard. Another kind of tools use risk-adjusted performance measurement (RAPM) to deal with the net monetary benefit (NMB), to determinate if the NMB is positive when taking the risk of expenses and outcomes as a kind of cost. For example, the cost‑effectiveness risk-aversion curve (CERAC, see Sendi [2]) uses the downside variance as a measure of risk and measuring the benefit-to-risk ratio as a RAPM to generate the curve. These tools present effective approaches for making medical decisions under uncertainty (see Al [3]). However, Sendi et al. [4] show that the most frequently used CEACs still have many weak points, as it is not sensitive to the changes of radial shifts of the joint distribution of incremental costs and effects in the North-East and South-West quadrants of the cost-effectiveness plane. Also, the level of risk aversion is not fully collaborated into these existing tools.Article structure: Kindly check and confirm whether the "Key points for decision makers" section is correctly processed. Yes，I have checked the section of key points for decision markers, I make sure that it is correctly processed.

Besides, other challenges confront the existing stochastic CEA methods. Correlation structure problems are noted by Barton et al. [5], showing that the correlation between the costs and outcomes will weaken the reliability of the conclusions of existing stochastic CEA tools. Also, it is mentioned that correlations of outcomes among different opinions from different individuals will also influence the judgment. Asymmetric uncertainty issues, as highlighted by Jakubczyk et al. [6], which can stem from small-sized samples, also could make troubles. This issue could cause the intervention with higher cost-effective probability to get a negative incremental net benefit. Moreover, the sub-optimality of a unique intervention, as mentioned in Sendi et al. [7], exists because of the heterogeneity of patients. The population of patients may consist of different etiologies or genotypes, and some are more suitable to the new intervention but some are not.

The challenges arising from correlation structures and the constraints from unique interventions can be addressed through the application of population benefits analysis tools. A population benefits analysis is to analysis the costs and outcomes, with consideration about risks and uncertainties, for a group of individuals instead of unique or typical ones. For the correlation structure problem, population benefits analysis can effectively differentiate the correlation of benefits for different patients within the same intervention and the correlation between different interventions’ outcomes and costs. Also, for the problem of the constraint of the unique intervention, a patient cannot choose to receive two interventions simultaneously, but a population of patients can choose to mix different interventions proportionally.

Furthermore, specific choices and assumptions in some methods have also been challenged in practical applications. Elbasha [8] compared the risk-adjusted performance measure of CERAC with other methods and found that choosing the Sortino ratio as a RAPM implies a highly risk-averse assumption, which causes the method to fail when the real level of risk aversion is not high. Kim et al. [9] also show that most studies lack proper considerations when selecting broader evaluation perspectives. Lomas [10] directly points out the non-marginality issue of existing methods, which may cause seemingly cost-effective interventions to be rejected in actual applications. A large proportion of the judgements are made under the current situation, which will only be reliable if the change is tiny enough. However, the suggestion of these judgements directly aims at further changes, which is out of the margin of the present one and hard to be reasonable. Paulden [11] challenges the function of traditional ICER as a “distance” by pointing out its impossibility on intervention ranking. These findings collectively suggest that a risk-adjusted ICER concerning on the benefits of the population with marginal judgements needs to be developed.

To this end, this paper proposes a stochastic cost-effectiveness analysis tool aiming at enhancing population benefits. We also present a discriminant tool for judging whether a medical decision changing an intervention to another for a small part of the population would contribute to this goal. Furthermore, we propose the analytical expressions for the discriminant measure in a traditional ICER form with good economic interpretation. Also, the properties and superiority of the proposed approach are shown with numerical simulations with some general assumptions on mean and covariance matrix. At last, the feasibility and excellence of the mixed interventions is verified by an empirical example.

## The optimization problem of population benefits

Population benefits refer to the total level of benefits for a specific group of patients. Different from the classical individual benefits problem, population benefits assess the total benefit level for all patients in the group. A group of patients with a specific condition, namely a patient population, may have different socio-demographic characteristics, may have different concomitant conditions, may have a different treatment history for the specific condition and may respond in different ways to the new intervention if they choose to try it. Therefore, population benefits analysis should be introduced to allow for the coexistence of different medical interventions and also takes the variability and correlation among the patients into account. The stochastic optimization problem aimed at the population benefits forces maximizing the overall population benefits by solving for the optimal structure of medical interventions while accounting for the patient heterogeneity.

### Model settings

Consider a specific population of patients, and the number of patients in the population is denoted as *M*. There are *N* optional interventions that may be effective for this population, with each intervention possessing potential or existing feasibility. Let *M*^*i*^ represent the number of patients in the group who have received the *i*th intervention, where $i \in \{ 1,2,\ldots,N\}$. Every patient requires an intervention, hence $\sum_{i = 1}^N {M^i } = M$. The vector ${{\mathbf{M}}} = \{ M^1 ,M^2 ,\ldots,M^N \}$, composed of all *M*^*i*^ values, represents the intervention structure adopted by this patient population. It is worth noting that some patients may not be taking any intervention, and “to take no intervention” is also a kind of intervention, named “Null Intervention” in our settings.

The net monetary benefit (NMB) is a measurement used to determine an individual's benefit level in monetary. This calculation employs a monetization coefficient *λ* to convert the quality-adjusted life-year (QALY) that a patient receives through a given intervention to a monetary value and forms a monetization return measure in summary. For the patient *m*^*i*^ who receives the *i*th intervention, their level of NMB, $X_{m^i }^i$, is defined as:

1 $$X_{m^i }^i : = \lambda {\text{QALY}}_{m^i }^i - {\text{Cost}}_{m^i }^i ,\quad\forall m^i \in \{ 1,2,\ldots,M^i \} ,\quad i \in \{ 1,2,\ldots,N\} .$$

As the costs incurred by patients during the intervention and their QALY outputs are stochastic, each $X_{m^i }^i$ represents a random variable. These random variables representing NMB levels belong to a random space, $(\Omega ,{{\mathcal{F}}},{{\mathbb{P}}})$. For any *i*, the expected value of $X_{m^i }^i$ is denoted as ${{\mathbb{E}}}[X_{m^i }^i ] = \mu^i$. It is generally assumed that $\{ X_{m^i }^i \}$ has cross-moments of any order, which means that one could calculate the moments of these variables in any orders.

Under a given intervention structure **M**, the population benefits *S*(**M**) of the studied population can be determined by summing up the NMB of each case. Therefore, the following definition is proposed.

#### Definition 1

(Population Benefits) We define the population benefits for the population of the patients with some specific conditions as a function $S( \cdot ):\;R^N \mapsto R$ that is *N*-dimensional. Specifically, given an intervention structure **M**, we express *S*(**M**) as:

2 $$S({{\mathbf{M}}}) = \sum_{i = 1}^N {\left( {\sum_{m^i = 1}^{M^i } {X_{m^i }^i } } \right)} .$$

### Optimization problem

The stochastic optimization problem for population benefits is to find the optimal intervention structure M*, which balances the expected benefits of the population and the risk level of them. This means that we want to get a higher population benefit in average, but do not want it to be too volitive. That could be done by optimizing a risk-adjusted performance measure on the population benefits. A risk-adjusted performance measure is a functional taking the average benefit and its uncertainty into consideration. It is monotonically increasing with respect to the expectation on benefits, but with risk penalties. Typical stochastic CEA studies use specific risk-adjusted performance measures, such as the Sharpe ratio and the Sortino ratio, to measure such trade-offs by pursuing expectation while controlling variance. This article employs a more generalized family of risk-adjusted performance measures, ${{\mathbb{E}}}[ \cdot ]/\rho ( \cdot )$, as the target functional of population benefits in risk-return trade-offs, where $\rho ( \cdot )$ is a one-dimensional non-negative functional used to represent the risk measure applied to the random variable of the population benefits (see Artzner et al. [12]). The risk measures commonly utilized in typical stochastic CEA research include variance (such as in the case of deterministic equivalence under the Constant Absolute Risk Aversion framework), standard deviation (such as in the case of the Sharpe Ratio), and downside deviation (in the case of the Sortino Ratio). Additionally, other risk measures such as value at risk (VaR) and conditional tail expectation (CTE) are employed in more generalized researches of risk.

Under the risk-adjusted performance measure, the target function of the stochastic optimization problem in this paper, which represents the risk-return ratio of population benefits, can be expressed as

3 $${{\mathbb{U}}}({{\mathbf{M}}}) = \frac{{{{\mathbb{E}}}(S({{\mathbf{M}}}))}}{{\rho (S({{\mathbf{M}}}))}}.$$

With this objective, subject to the constraint $\sum_{i = 1}^N {M^i } = M$, the stochastic optimization problem can be represented as:

4 $${\mathop {\max }\limits_{{\mathbf{M}}}} \;{{\mathbb{U}}}({{\mathbf{M}}}),\quad s.t.\quad \frac{{\left\| {{\mathbf{M}}} \right\|_1 }}{M} = 1.$$

Similar to the discussion in Buch et al. [13], when *M* is sufficiently large, the risk measure is sub-additive, and $\rho^{ij} \in (0,1),\forall i,j$, the optimization problem (4) has a unique positive inner solution, denoted as M*. This inner solution represents the optimal intervention structure that maximizes the risk-return ratio of population benefits.

### The necessary conditions for the optimal solution of the problem

Assuming that *M* is sufficiently large, we can assert that ${{\mathbb{U}}}({{\mathbf{M}}} + {{\mathbf{e}}}^i ) - {{\mathbb{U}}}({{\mathbf{M}}})$ can be approximated by $\partial {{\mathbb{U}}}({{\mathbf{M}}})/\partial M^i$, where **e**^*i*^ is an *N*-dimensional basis vector with the *i*th element being 1 and the remaining elements being 0. Under this situation, the optimization problem (4) can be solved using the Lagrange method. Specifically, the following theorem specifies the necessary conditions that the optimal intervention structure should satisfy.

#### Theorem 1

*Assuming that M is sufficiently large, the inner solution for the optimization problem expressed in formula* (4), *which is the optimal intervention structure M, must satisfy the following necessary conditions*:

5 $$\mu^i \rho (S({{\mathbf{M}}})) - {{\mathbb{E}}}[S({{\mathbf{M}}})]\frac{{\partial \rho (S({{\mathbf{M}}}))}}{\partial M^i } = \gamma \rho^2 (S({{\mathbf{M}}})),\quad \forall i \in \{ 1,2,\ldots,N\} ,$$

*where* *γ* *is a non-zero constant*.

#### Proof of Theorem 1

For Eq. (4), We construct the Lagrange function,

6 $${{\mathcal{L}}}({{\mathbf{M}}},\gamma ) = \frac{{{{\mathbb{E}}}(S({{\mathbf{M}}}))}}{{\rho (S({{\mathbf{M}}}))}} - \gamma \left( {\sum_{i = 1}^N {M^i } - M} \right),$$

where *γ* is the Lagrange parameter which must be non-zero due to the nature of the constraints. Thus, the inner solution must satisfy the condition $\partial {{\mathcal{L}}}({{\mathbf{M}}},\gamma )/\partial M^i = 0$ for any *i*, then we can get

7 $$\frac{\mu^i }{{\rho (S({{\mathbf{M}}}))}} - \frac{{{{\mathbb{E}}}[S({{\mathbf{M}}})]}}{{\rho^2 (S({{\mathbf{M}}}))}}\frac{{\partial \rho (S({{\mathbf{M}}}))}}{\partial M^i } - r = 0,\quad \forall i,$$

which are necessary conditions. Moreover, the properties of risk measures stipulate that $\rho (S({{\mathbf{M}}})) \ne 0$, then we get Eq. (5). □

Theorem 1 shows that under certain conditions, the optimal intervention structure can be obtained by solving the system of equations composed of Eq. (5) and $\sum_{i = 1}^N {M^i } = M$. For medical decision-making situations with adequate decision independence, selecting the intervention structure derived from solving these equations can lead to the maximum risk-return ratio of the population benefits.

## Stochastic cost-effectiveness analysis

In general, the medical decision-making is often limited by various internal and external factors. It is challenging for decision-makers to fully reconstruct the optimal intervention structure of the group within a single period. To face this situation, we introduce the stochastic cost-effectiveness criterion for population benefits for that any two interventions could be compared. One can also find the similarities and differences between the tools in this paper and traditional CEA from the criterion.

From a population perspective, the superiority of one intervention over another can be understood in a form of marginal contribution. In other words, if the intervention for a unit of patients switches from one to another and the RAPM of the population benefits increase, then the latter intervention can be considered to be marginally superior to the former. With the model framework and theorem presented earlier, we can obtain the marginal optimization theorem for population benefits, which enables us to determine whether one of any two interventions satisfies the superiority criterion for stochastic CEA over the other under the current situation.

### Marginal optimization theorem for population benefits

The marginal criterion for stochastic CEA on population benefits can be expressed through the following theorem.

#### Theorem 2

(Marginal optimization theorem for population benefits): *The equivalence criterion for an intervention to dominate another in a population sense is that the additional risk-adjusted expected cost to obtain a unit increase in expected QALY is less than the monetization factor* $\lambda$.

*Mathematically, for any* $i,j \in \{ 1,2,\ldots,N\}$, *if* ${{\mathbb{E}}}[{\text{QALY}}^j ] - {{\mathbb{E}}}[{\text{QALY}}^i ] > 0$, *then*

8 $${\mathop {\lim }\limits_{c \to 0^+ }} \frac{{{{\mathbb{U}}}({{\mathbf{M}}} - c{{\mathbf{e}}}^i + c{{\mathbf{e}}}^j ) - {{\mathbb{U}}}({{\mathbf{M}}})}}{c} > 0 \Leftrightarrow \frac{{{{\mathbb{E}}}^Q [{\text{Cost}}^j ] - {{\mathbb{E}}}^Q [{\text{Cost}}^i ]}}{{{{\mathbb{E}}}[{\text{QALY}}^j ] - {{\mathbb{E}}}[{\text{QALY}}^i ]}} < \lambda ,$$

*where* ${{\mathbb{E}}}^Q [{\text{Cost}}^j ]$ *is the risk-adjusted expected cost under the intervention* *j*, *defined as*

9 $${{\mathbb{E}}}^Q [{\text{Cost}}^j ] = {{\mathbb{E}}}[{\text{Cost}}^j ] + \alpha ({{\mathbf{M}}})\frac{{\partial \rho (S({{\mathbf{M}}}))}}{\partial M^j },$$

*and* $\alpha ({{\mathbf{M}}}) = {{\mathbb{E}}}(S({{\mathbf{M}}}))/\rho (S({{\mathbf{M}}}))$ *is the current level of risk-return for population benefits*.

#### Proof of Theorem 2

First, the substitution definition, $D(c) = {{\mathbb{U}}}({{\mathbf{M}}} - c{{\mathbf{e}}}^i + c{{\mathbf{e}}}^j )$, denotes the return-to-risk level of population benefits after transferring patients of an amount of *c* from intervention *i* to intervention *j*. By the definition of partial derivatives, $D( \cdot )$’s continuity for *c* and the uniform integrability of *S*(**M**), there is

10 $${\mathop {\lim }\limits_{c \to 0^+ }} \frac{\partial D(c)}{{\partial c}} = {\mathop {\lim }\limits_{c \to 0^+ }} {\mathop {\lim }\limits_{\Delta c \to 0^+ }} \frac{D(c + \Delta c) - D(c)}{{\Delta c}} = {\mathop {\lim }\limits_{c \to 0^+ }} \frac{{{{\mathbb{U}}}({{\mathbf{M}}} - c{{\mathbf{e}}}^i + c{{\mathbf{e}}}^j ) - {{\mathbb{U}}}({{\mathbf{M}}})}}{c},$$

and then only the equivalence condition of ${\mathop {\lim }\limits_{c \to 0^+ }} \frac{\partial D(c)}{{\partial c}} > 0$ needs to be examined.

Since we have

11 $$\frac{\partial D(c)}{{\partial c}} = \frac{{{{\mathbb{U}}}({{\mathbf{M}}} - c{{\mathbf{e}}}^i + c{{\mathbf{e}}}^j )}}{\partial M^i }\frac{\partial (M^i - c)}{{\partial c}} + \frac{{{{\mathbb{U}}}({{\mathbf{M}}} - c{{\mathbf{e}}}^i + c{{\mathbf{e}}}^j )}}{\partial M^j }\frac{\partial (M^j + c)}{{\partial c}},$$

we can obtain

12 $$\begin{aligned} {\mathop {\lim }\limits_{c \to 0^+ }} \frac{\partial D(c)}{{\partial c}} &= - \frac{{\partial {{\mathbb{U}}}({{\mathbf{M}}})}}{\partial M^i } + \frac{{\partial {{\mathbb{U}}}({{\mathbf{M}}})}}{\partial M^j } \\ &= - \frac{\mu^i }{{\rho (S({{\mathbf{M}}}))}} + \frac{{{{\mathbb{E}}}[S({{\mathbf{M}}})]}}{{\rho^2 (S({{\mathbf{M}}}))}}\frac{{\partial \rho (S({{\mathbf{M}}}))}}{\partial M^i } + \frac{\mu^j }{{\rho (S({{\mathbf{M}}}))}} - \frac{{{{\mathbb{E}}}[S({{\mathbf{M}}})]}}{{\rho^2 (S({{\mathbf{M}}}))}}\frac{{\partial \rho (S({{\mathbf{M}}}))}}{\partial M^j } \\ &= \frac{1}{{\rho (S({{\mathbf{M}}}))}}\left( {\mu^j - \mu^i - \frac{{{{\mathbb{E}}}[S({{\mathbf{M}}})]}}{{\rho (S({{\mathbf{M}}}))}}\left( {\frac{{\partial \rho (S({{\mathbf{M}}}))}}{\partial M^j } - \frac{{\partial \rho (S({{\mathbf{M}}}))}}{\partial M^i }} \right)} \right) \\ &= \frac{1}{{\rho (S({{\mathbf{M}}}))}}\left( {\mu^j - \mu^i - \alpha ({{\mathbf{M}}})\left( {\frac{{\partial \rho (S({{\mathbf{M}}}))}}{\partial M^j } - \frac{{\partial \rho (S({{\mathbf{M}}}))}}{\partial M^i }} \right)} \right). \\ \end{aligned}$$

Since $\rho (S({{\mathbf{M}}})) > 0$, the equivalence condition for ${\mathop {\lim }\limits_{c \to 0^+ }} \frac{\partial D(c)}{{\partial c}} > 0$ can be expressed as

13 $$\mu^j - \mu^i - \alpha ({{\mathbf{M}}})\left( {\frac{{\partial \rho (S({{\mathbf{M}}}))}}{\partial M^j } - \frac{{\partial \rho (S({{\mathbf{M}}}))}}{\partial M^i }} \right) > 0,$$

and there is $\forall i,\quad \mu^i = \lambda {{\mathbb{E}}}[{\text{QALY}}^i ] - {{\mathbb{E}}}[{\text{Cost}}^i ]$, which is substituted to express the equivalence condition as

14 $$\lambda {{\mathbb{E}}}[{\text{QALY}}^j ] - \lambda {{\mathbb{E}}}[{\text{QALY}}^i ] - {{\mathbb{E}}}[{\text{Cost}}^j ] + {{\mathbb{E}}}[{\text{Cost}}^i ] - \alpha ({{\mathbf{M}}})\left( {\frac{{\partial \rho (S({{\mathbf{M}}}))}}{\partial M^j } - \frac{{\partial \rho (S({{\mathbf{M}}}))}}{\partial M^i }} \right) > 0.$$

Noting that

15 $${{\mathbb{E}}}^Q [{\text{Cost}}^j ] = {{\mathbb{E}}}[{\text{Cost}}^j ] + \alpha ({{\mathbf{M}}})\frac{{\partial \rho (S({{\mathbf{M}}}))}}{\partial M^j },$$

there is

16 $$\lambda \left( {{{\mathbb{E}}}[{\text{QALY}}^j ] - {{\mathbb{E}}}[{\text{QALY}}^i ]} \right) > {{\mathbb{E}}}^Q [{\text{Cost}}^j ] - {{\mathbb{E}}}^Q [{\text{Cost}}^i ],$$

then the theorem is proved with ${{\mathbb{E}}}[{\text{QALY}}^j ] - {{\mathbb{E}}}[{\text{QALY}}^i ] > 0$. □

It can be seen that Theorem 2 provides a way of comparing the marginal cost-effectiveness of any two interventions when considering uncertainty. The expression on the left-hand side of Eq. (8) measures the change in the risk-return ratio of population benefits when switching from intervention *i* to intervention *j* on a small unit of patients. The expression on the right-hand side of Eq. (8) measures the incremental risk-adjusted expected cost required to achieve one unit of incremental QALY, by transforming from intervention *i* to intervention *j*. In other words, under the current intervention structure, an intervention switch results in an incremental risk-adjusted expected cost ratio for gaining incremental QALY less than $\lambda$ is beneficial to the risk-return ratio of population benefits.

It is worth noting that although the criterion in Theorem 2 has a form similar to that of classical ICER, the main difference lies in the measurement of “expected costs”. Here, the incremental expected cost needs to be measured from the perspective of risk adjustment, that is, considering the “implicit” adjustment corresponding to the risk. In the definition formula, Eq. (9), of the risk-adjusted expected cost, *α*(**M**) is the risk-return level for population benefits under the current intervention structure, which can be understood as the current return-to-risk rate. The $\partial \rho (S({{\mathbf{M}}}))/\partial M^j$ is a risk allocation method (often called a marginal allocation, see Denault [14]) and can be understood as the risk level allocated to one unit of patients receiving intervention *j* when the risk measure of the population benefits is $\rho (S({{\mathbf{M}}}))$. It is worth noting that although it is named as “risk-adjusted expected cost”, it does not mean that the adjustment only considers the risk in costs. Instead, all risks and uncertainties are taking into consideration, containing the risks of costs and outcomes. The name is just because the adjustment is made on the term of cost. Furthermore, the risk-adjusted expected cost can be understood as the sum of the expected cost (used in classical CEAs) and the risk cost allocated to the intervention. And then, the criteria are constructed using such risk-adjusted expected costs.

### Risk-adjusted ICER

The criterion in Theorem 2 uses a specific form of ICER as a measure to discriminant the superiority of any two interventions measured under the consideration of stochasticity. Such a criterion is defined as the risk-adjusted ICER due to the utilization of risk-adjusted expected costs.

#### Definition 2

(Risk-adjusted ICER): Under the intervention structure **M**, if ${{\mathbb{E}}}[{\text{QALY}}^j ] - {{\mathbb{E}}}[{\text{QALY}}^i ] > 0$, the risk-adjusted ICER, ${\text{ICER}}_{i,j}^Q ({{\mathbf{M}}})$, of switching intervention from *i* to *j*, is defined as:

17 $${\text{ICER}}_{i,j}^Q ({{\mathbf{M}}}) = \frac{{{{\mathbb{E}}}^Q [{\text{Cost}}^j ] - {{\mathbb{E}}}^Q [{\text{Cost}}^i ]}}{{{{\mathbb{E}}}[{\text{QALY}}^j ] - {{\mathbb{E}}}[{\text{QALY}}^i ]}},$$

where ${{\mathbb{E}}}^Q [{\text{Cost}}^j ]$ is the **Risk-Adjusted Expected Cost** under intervention *j*, defined as:

18 $${{\mathbb{E}}}^Q [{\text{Cost}}^j ] = {{\mathbb{E}}}[{\text{Cost}}^j ] + \alpha ({{\mathbf{M}}})\frac{{\partial \rho (S({{\mathbf{M}}}))}}{\partial M^j },$$

and $\alpha ({{\mathbf{M}}}) = {{\mathbb{E}}}(S({{\mathbf{M}}}))/\rho (S({{\mathbf{M}}}))$ is the risk-return level of the current population benefits.

It can be seen that risk-adjusted ICER is an incremental cost-effectiveness ratio based on the risk-adjusted expected cost. Therefore, risk-adjusted ICER is a generalization of classical ICER considering all sources of risks. Compared with other CEA methods that consider stochasticity, risk-adjusted ICER has several typical advantages. First, from the perspective of the risk-return ratio of population benefits, risk-adjusted ICER allows different interventions to coexist, solving the “either black or white” challenge faced by classical CEA for individuals. Second, risk-adjusted ICER is a marginal discriminant condition that incorporates the current intervention structure into the decision of optimality, allowing medical decision-makers to gradually adjust interventions. This means that the decision is made under the current situation, which ensured the reliability. Third, the risk-adjusted ICER accommodates any degree of risk aversion in the risk measure selection, allowing medical decision-makers to select any risk measure for CEA analysis without the need for special discriminant tools for each measure. Finally, risk-adjusted ICER retains the classical ICER form, and the monetization constant $\lambda$ remains unchanged from the original setting, ensuring the explicit distinction between cost and QALY in the expressions.

Technically, it is worth adding the condition, ${{\mathbb{E}}}[{\text{QALY}}^j ] - {{\mathbb{E}}}[{\text{QALY}}^i ] > 0$, which actually only requires a specified order of intervention *i* and intervention *j* according to their ${{\mathbb{E}}}[{\text{QALY}}]$ values when comparing the two interventions. Indeed, excluding interventions that are exactly equivalent in expected QALY, the set of situations with superiority of *j* over *i* and situations of *i* over *j* are complementary. The decision result can be obtained by comparing in any determined order. In other word, one can always choose the intervention with larger expected QALY to be the intervention j, and use these two Theorem to may decisions. This means that the condition is without loss of generality, and therefore, both Theorems 1 and 2 are stated under a specified order, and they also have equivalent dual forms in the other order, which will not be repeated here.

## Numerical and empirical examples

In order to further clarify the properties of the method proposed in this paper and demonstrate its association and difference with classical CEA tools, we perform numerical simulations to calculate the results, differences, and effectiveness of various types of uncertain CEA tools. Further, we take an empirical example to show that the mixed structure of interventions could get a higher population benefit, and the tool in this paper helps to find it.

### Stochastic system settings

We choose the mean–variance system to describe the stochasticity of the simulation system to simply and clarify the simulation processes and results. It is assumed that the joint distribution of the costs and QALY outcomes of individuals receiving various interventions follows an overall joint multinormal distribution. Therefore, characterizing all the stochasticity can be accomplished by setting the means, standard deviations, and the matrix of the correlation coefficient. For each intervention, it is assumed that the costs and QALY outcomes of each patient taking this intervention are identically distributed but not independent. And, there is a certain correlation between the costs and QALY outcomes of individual patients. The correlation between patient costs of different interventions is only related to the intervention they take, but without distinguishing individuals. Same assumption applies to QALY outcomes. In addition, it is assumed that the cost of any patient is independent of the QALY outcome of other patients, which means that the cost of patient A does not correlate with the QALY outcome of any patient B.

Correlation matrix can express the structure of correlation much clearer. Specifically, for each group of patients taking the same intervention, the costs and QALY outcomes of each patient have unique mean and variance values. That is, for any *i* and *m*^*i*^, we have

19 $$\begin{aligned}& {{\mathbb{E}}}[{\text{QALY}}_{m^i }^i ] = \mu_Q^i ,\;{\text{ Var}}[{\text{QALY}}_{m^i }^i ] = (\sigma_Q^i )^2 {{\ominus }}, \\ &{{\mathbb{E}}}[{\text{Cost}}_{m^i }^i ] = \mu_C^i ,\quad\; {\text{ Var}}[{\text{Cost}}_{m^i }^i ] = (\sigma_C^i )^2 . \hfill \\ \end{aligned}$$

And for the correlation coefficient of each cost, there is

20 $$\forall i,j,\;{\text{Corr}}[{\text{Cost}}_{m_1 }^i ,{\text{Cost}}_{m_2 }^j ] = \left\{ {\begin{array}{ll} {1,} &\quad {i = j,\;m_1 = m_2 } \\ {\rho_C^{ii} ,} &\quad {i = j,\;m_1 \ne m_2 } \\ {\rho_C^{ij} ,} &\quad {i \ne j,} \\ \end{array} } \right.$$

and for each QALY there is a similar equation

21 $$\forall i,j,\;{\text{Corr}}[{\text{QALY}}_{m_1 }^i ,{\text{QALY}}_{m_2 }^j ] = \left\{ {\begin{array}{ll} {1,} &\quad {i = j,\;m_1 = m_2 } \\ {\rho_Q^{ii} ,} &\quad {i = j,\;m_1 \ne m_2 } \\ {\rho_Q^{ij} ,} &\quad {i \ne j.} \\ \end{array} } \right. \,$$

In addition, the cross-sectional relationship between cost and QALY output is assumed as

22 $$\forall i,j,\;{\text{Corr}}[{\text{Cost}}_{m_1 }^i ,{\text{QALY}}_{m_2 }^j ] = \left\{ {\begin{array}{ll} {\rho_{QC}^i ,} &\quad {i = j,\;m_1 = m_2 } \\ {\rho_{QC}^{ii} = 0,} &\quad {i = j,\;m_1 \ne m_2 } \\ {\rho_{QC}^{ij} = 0,} &\quad {i \ne j.} \\ \end{array} } \right.$$

**Fig. 1:**
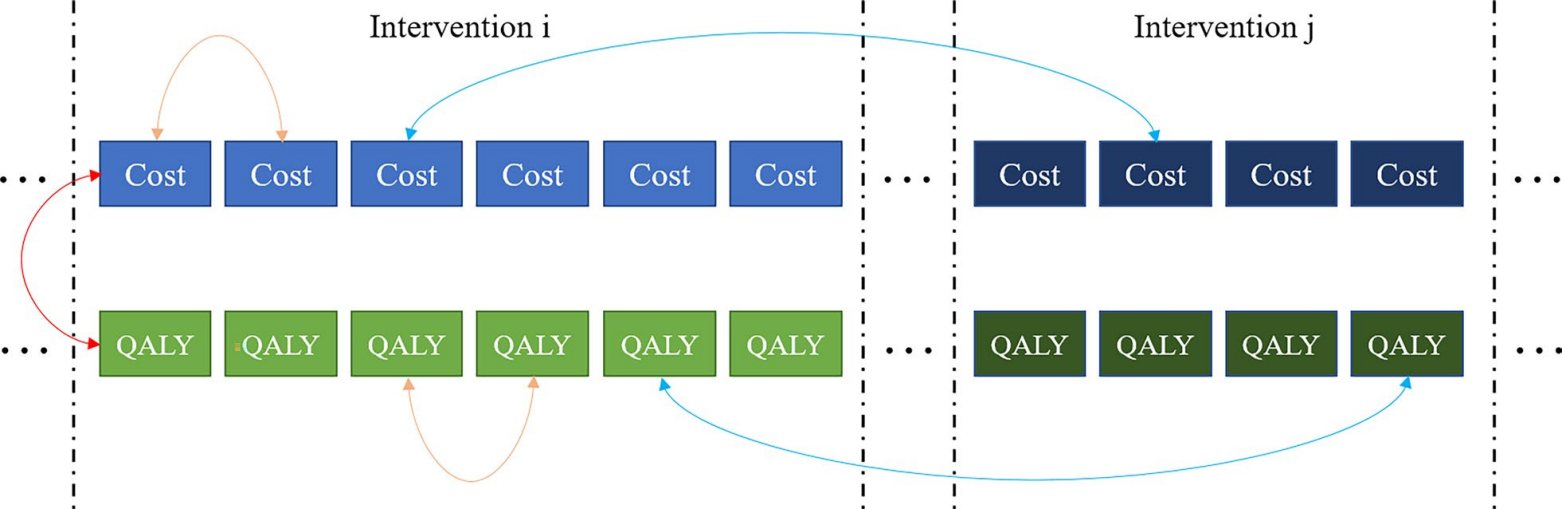
The correlation between different random variables

The structure of each correlation is schematically shown in the following Fig. 1.

### Simulation methods and settings

Given a selected intervention structure, the numerical simulation uses Monte Carlo sampling to form a column vector that combines the costs and QALY outputs of each patient under the assumed parameters. A sufficient number of samples are generated and the statistical features of the overall results (such as population benefits and total costs) are calculated. Then, the results of the objective function under the given intervention structure are obtained. Following this, different stochastic CEA methods are applied to this problem to get their own judgements on optimal interventions. Performance of population benefits are compared with these optimal interventions. Moreover, simulations are performed again for each method's recommended intervention structure modification, showing the effect of recommendations for different stochastic CEA methods’ judgements. Some key intermediate results are also recorded and presented.

#### Simulation process

The purpose of simulation mainly lies in three aspects. Firstly, illustrating through numerical examples that, in terms of population benefits, the mixture of different interventions could have a better return-to-risk result than an absolutely unique intervention. Secondly, under different intervention structures, calculating the risk-adjusted expected cost and risk-adjusted ICER proposed in this paper, and verifying the process of inducing the optimal risk-return ratio of population benefits in a marginal sense. Thirdly, comparing the decisions generated by different stochastic CEA methods for the same problem, testing the performance of different methods on population benefits, and checking their suggested direction of optimization.

Specifically, we first select two interventions from all optional ones, separately calculate their risk-return levels of population benefits, and then mixes them in different proportions to form intervention structures. Secondly, under different intervention structures, the risk-adjusted expected cost and risk-adjusted ICER of the two interventions are calculated, along with the corresponding decisions that the criterion makes for optimizing the target function. Then, to show the effectiveness of each method, a specific intervention structure is selected, and different stochastic CEAs are used for making determinations between any two interventions. The changes in the performance of population benefits are measured after adjusting the intervention structure according to the corresponding determination. These changes can show the effectiveness of different methods.

In the simulation process, it is necessary to simulate the QALY outcomes and costs under the selected intervention and evaluate the population benefits quantitatively. In detail, under each selected intervention structure **M**, the evaluation of the return-to-risk ratio of the population benefits level consists of the following steps in the simulation process:

1. Divide the *M* simulated patients into *N* groups, where the *i*th group has *M*^*i*^ patients according to the structure of **M**.
2. Combine the random variables of cost and QALY output of each patient into a random vector and calculate the mean and covariance matrix of the random vector.
3. Generate 10,000 samples of the group using the calculated mean and covariance matrix as the parameters of a high-dimensional multinormal distribution.
4. Measure the population benefits of each sample of the group.
5. Summarize the population benefits of each sample and measure their statistical characteristics and return-to-risk ratios.

Since the simulation can only generate a limited number of samples, selecting extremely tailed risk measures may cause the simulation results to be unstable. Therefore, we repeat the evaluating steps mentioned above 100 times independently and use the average value as the final result. Finally, we use empirical probability of correctly recommendations to estimate the accuracy of different CEAs.

#### Parameter settings and reference methods selection

To balance the adequacy of alternative interventions and the interpretability of results, we select a total of *N* = 5 interventions and assume that the patient population consists of *M* = 100 individuals. The number of samplings of the outcome results of any patient subgroup is chosen to be 10,000, and the number of repeated experiments for the return-to-risk evaluation of population benefits is chosen to be 100 times.

The assumed distribution parameters within intervention subgroups are shown in Table 1:

**Table 1:** Assumptions of distribution parameters within intervention subgroups

${\text{ID (i)}}$	$\mu_Q^i$	$\mu_C^i$	$\sigma_Q^i$	$\sigma_C^i$	$\rho_Q^{ii}$	$\rho_C^{ii}$	$\rho_{QC}^{ii}$
1	7.5	6000	0.1	100	0.03	0.04	0.02
2	9	8000	0.05	200	0.04	0.04	0.02
3	10	10,000	0.1	300	0.03	0.03	0.02
4	12	12,000	0.15	400	0.03	0.03	0.02
5	14	15,000	0.2	200	0.03	0.03	0.02

In addition, the correlation coefficients between different individual outcomes among different groups are set to $\forall i \ne j,\;\rho_C^{ij} = 0.005,\;\rho_Q^{ij} = 0.002$, and the monetary constant, $\lambda$, is set to $\lambda = 2000\;\$ /{\text{QALY}}$ unless specifically mentioned.

Besides the method proposed in this paper, we select four different stochastic CEA methods as reference methods to participate in the evaluation together. The first is the classical ICER method, where the criterion measure is

23 $${\text{ICER}}_{i,j} ({{\mathbf{M}}}) = \frac{{{{\mathbb{E}}}[{\text{Cost}}^j ] - {{\mathbb{E}}}[{\text{Cost}}^i ]}}{{{{\mathbb{E}}}[{\text{QALY}}^j ] - {{\mathbb{E}}}[{\text{QALY}}^i ]}}.$$

The second is the Sortino ratio method (see Elbasha [8]) based on NMB, which compares the Sortino ratios (a kind of return-to-risk ratio using the expected benefit dividend by the downside variance) of different interventions to determine their priority. The third and fourth methods are the probabilistic methods based on CEAC, which determine the superiority of the interventions by judging whether the *α* percentile of the ICER distribution, $Q_\alpha (\frac{{{\text{Cost}}^j - {\text{Cost}}^i }}{{{\text{QALY}}^j - {\text{QALY}}^i }})$, satisfies the requirement of the willing-to-pay level ($\lambda$). The third method is relatively aggressive, using *α* = 5%, while the fourth method is relatively conservative, using *α* = 95%. Comparisons are made between the method proposed in this paper and these four reference methods.

### Simulation results

The simulation results are mainly presented in three parts. The first part shows the superiority of mixed intervention compared to unique intervention from the perspective of population benefits. The second part displays the relationship between changes in the risk-adjusted ICER and the intervention structures, which demonstrates the marginal optimality under different intervention structures. The third part shows the preference decisions of different interventions by different stochastic CEA methods under specific intervention structures, which demonstrates the validity of the decision by the tool of this paper.

#### Population benefits with mixed intervention

**Fig. 2:**
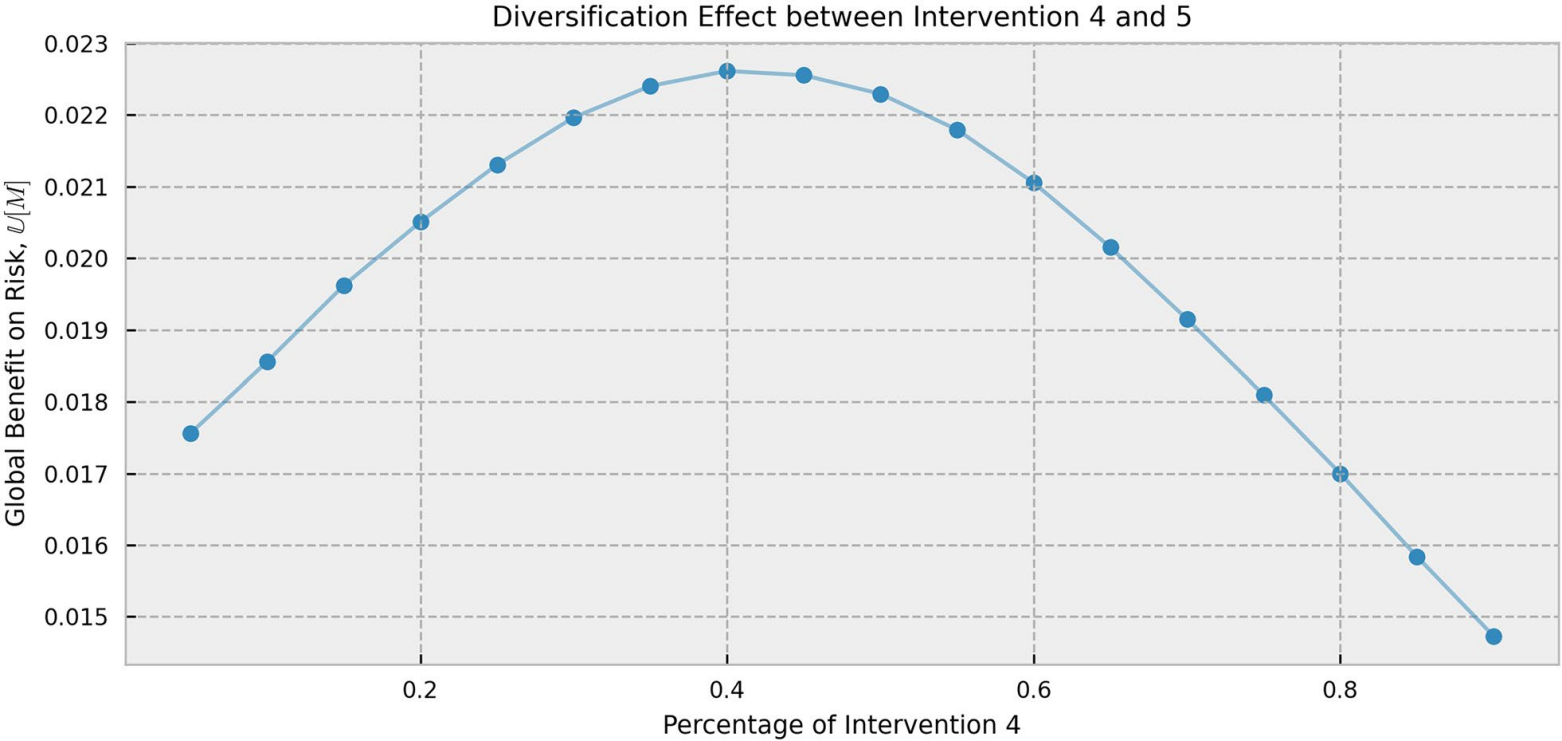
Effects on the population benefits of different intervention structures

In this subsection, we evaluate the population benefits under different intervention structures through simulation, and the incremental population benefits introduced by intervention mixing. It is verified that mixed interventions can lead to better return-to-risk ratios. Specifically, intervention 4 and intervention 5 are chosen as the controlled subgroups of this part, while the first three interventions have proportions of 0 in the structure, i.e., *M*^1^, *M*^2^, *M*^3^ = 0 to avoid extra interference. Then, the level of *M*^4^/*M* gradually varies from 0 to 1, and the corresponding return-to-risk ratios of the population benefits are calculated. The results are shown in Fig. 2.

From Fig. 2, it can be seen that as the proportion of intervention 4 gradually increases from 0 to 1, the return-of-risk ratios of population benefits shows a concave pattern, which firstly increasing and then decreasing. The target function reaches its optimum at around *M*^4^/*M* = 0.4. The optimum level, 0.0227, is significantly higher than the return-of-risk ratio of 0.0173 at *M*^4^/*M* = 0 and 0.0127 at *M*^4^/*M* = 1. This suggests that if considering the medical decisions with intervention 4 and intervention 5, using a 4:6 structure to mix both interventions is more efficient in a population view than using either intervention alone. This validates that it can be superior to mix interventions for population benefits.

#### Risk-adjusted expected cost and risk-adjusted ICER

**Fig. 3:**
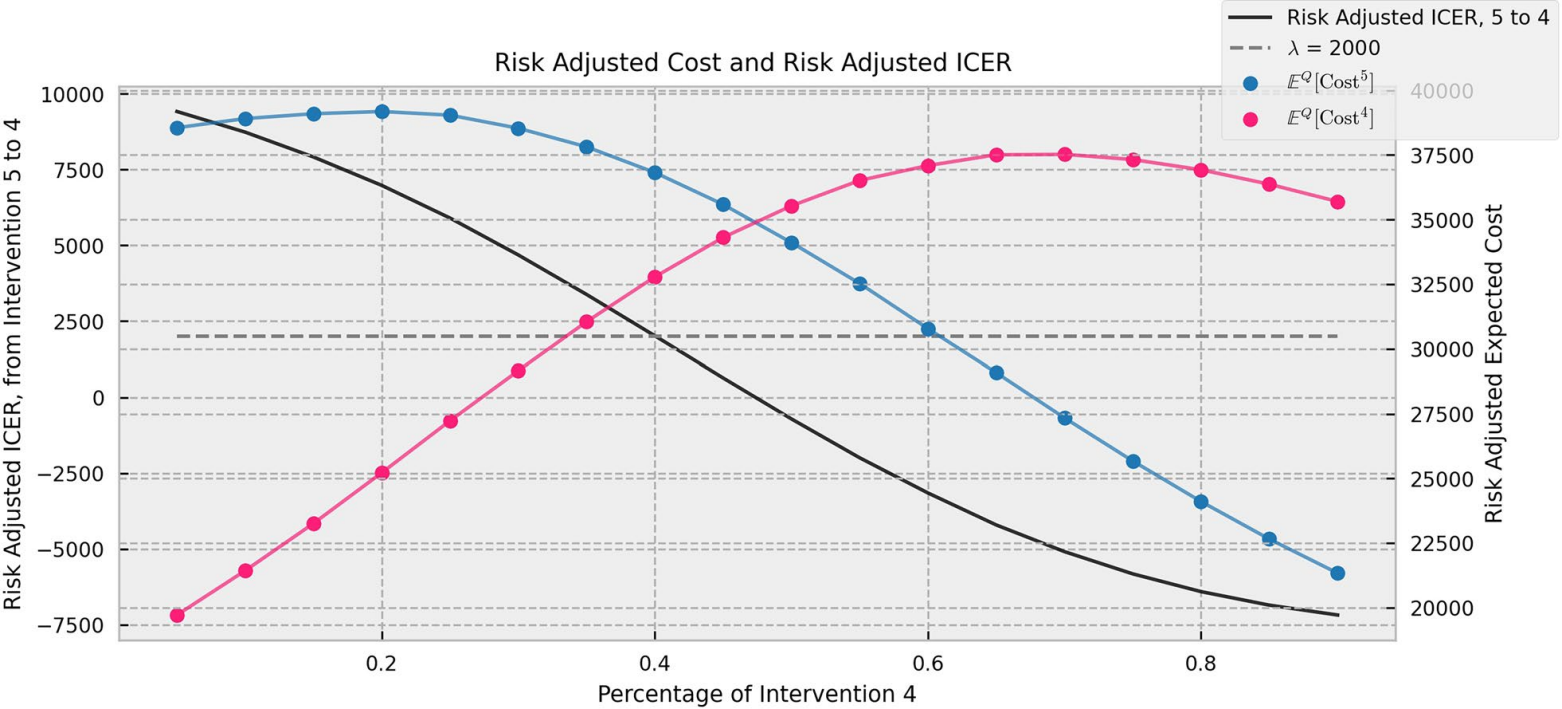
Risk-adjusted expected cost and risk-adjusted ICER for different structures

Following the previous subsection, we calculate risk-adjusted expected cost of both interventions and the risk-adjusted ICER for switching from intervention 4 to intervention 5 marginally as we gradually adjust the proportion of *M*^4^/*M* in the [0, 1] range. Through the simulation results, it is shown that the risk-adjusted ICER changes can lead the intervention structure to achieve the optimum. The simulation results are shown in Fig. 3.

In Fig. 3, the red curve represents the risk-adjusted expected cost of intervention 4, and the blue curve represents that of intervention 5. It can be seen that as the proportion of intervention 4 increases, the risk-adjusted expected costs’ difference between intervention 5 and intervention 4 changes from extremely positive to extremely negative. This is caused by changes in the diversification structure. Specifically, when the proportion of intervention 4 is small, there are many cases using intervention 5 in the population. At this time, increasing one unit of intervention 5 cases is equivalent to adding a highly positive correlated risk to the total risk pool, resulting in a relatively high level of risk allocated to the newly added one. This makes the risk-adjusted expected cost of the new unit of intervention 5 high at this situation. In contrast, the risk-adjusted expected cost of intervention 4 is relatively low because it is relatively independent with the risk pool. This pattern validates that the risk-adjusted expected cost does take the marginal impact of intervention restructuring on the risk pool into account in the risk adjustment.

In addition, the black curve in Fig. 3 represents the evaluation results of the risk-adjusted ICER from intervention 4 to 5. It can be seen that this curve intersects with the $\lambda = 2000$ threshold at around *M*^4^/*M* = 0.4, the optimal structure point. To the left of the intersection point, the risk-adjusted ICER is higher than the monetized constant, indicating that switching one unit of patients from intervention 4 to intervention 5 is not cost-effective and has a negative impact on the population benefits. Therefore, the proportion of intervention 5 needs to be reduced. On the contrary, to the right of the intersection point, the opposite is true. It is worth noting that the intersection of the black curve in Fig. 3 with $\lambda = 2000$ coincides perfectly with the position where the return-of-risk ratio of population benefits gets its optimum in Fig. 2. This also confirms that the risk-adjusted ICER can induce changes in the intervention structure to achieve the optimum.

#### Comparison of methods

**Fig. 4:**
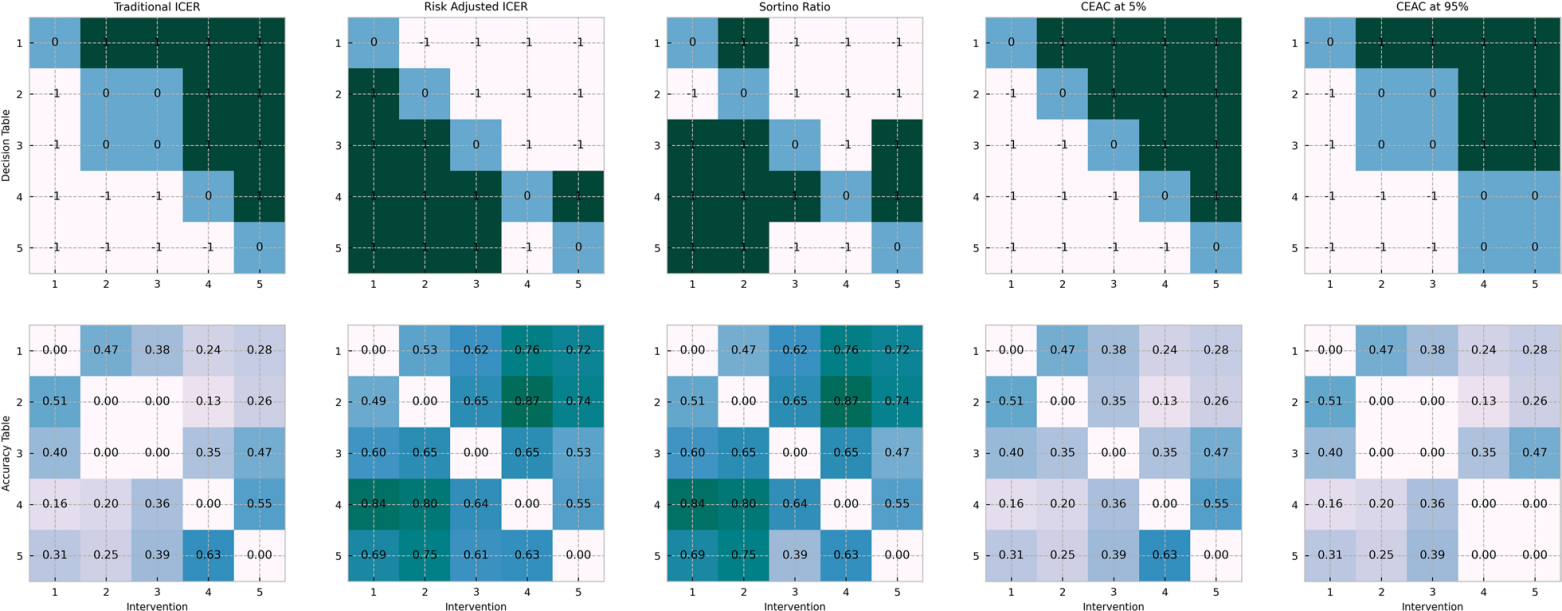
Decisions and correctness of various stochastic CEA methods

In this subsection, we selected a specific intervention structure (equal-weighted) as the starting point to examine the decisions of different stochastic CEAs for adjusting interventions and quantified the corresponding impact on the distribution of the population benefits. These results are shown in Fig. 4.

“Traditional ICER” represents the results of the traditional ICER tool, “Risk-adjusted ICER” represents our proposed method, “Sortino Ratio” represents the decision made using the Sortino ratio tool, “CEAC at 5%” represents the aggressive CEAC method, and “CEAC at 95%” represents the conservative CEAC method.

In Fig. 4, each sub-plot presents the information in a 5 × 5 matrix, where the grid in the *i*th row and *j*th column of any sub-plot represents the decision made by the corresponding stochastic CEA tools or its correctness, for changing an unit of patients from intervention *i* to intervention *j*. The decisions are shown in the sub-plots of the first row, where + 1 represents a decision to accept the change, − 1 represents a decision to accept an inverse change, and 0 represents no modification. The correctness of the decision results is displayed in the sub-plots of the second row, where the number in each grid represents the percentage of scenarios where population benefits are improved after the changes are taken based on the corresponding decision, within the 100 times of simulated scenarios. Additionally, the cells on the diagonal of each sub-plot do not contain any information.

From this comparison, it can be seen that the decisions of the traditional ICER and CEAC methods differ from our proposed method and the Sortino method to some extent. Specifically, the traditional ICER does not consider the stochasticity and only requires the ICER to be below the monetized constant in an expected view for each accepted intervention change. And, the CEAC method, similar to the traditional ICER, does not use the information contained in the current intervention structure. The Sortino Ratio method obtained similar decisions with respect to our proposed method, which can be attributed to the similarity in risk-adjusted measures between the two methods. However, the Sortino Ratio method still does not consider the current intervention structure, so there are still differences. These differences are not very significant due to the low level of correlation assumed in the simulation.

Also, it can be found that only our proposed method can almost perfectly ensure that the decisions to achieve an accuracy over 50%, which also reaches the highest level among all methods in expectation (The absolute value of the accuracy is related to the number of simulated samples, so the relative level of the accuracy of each method is mainly compared.). In addition, except for the judgement^1^ between intervention 2 and 1, our proposed method achieved the highest accuracy rate among all methods for any pair of comparisons. This also validates the effectiveness of our proposed method.

### An empirical example

In this subsection, we discuss an empirical example using the information extracted from recent research on comparing direct oral anticoagulants compared to low-molecular-weight-heparins for treatment of cancer associated venous thromboembolism, see Muñoz et al. [15]. It is worth noting that we just use some of the parameters from the research, but do not try to answer or challenge any problem or conclusion about this health technology assessing problem in real world. For short, we just borrow some parameters from this work to show that the population can benefit from mixed interventions, and we can find the optimal mix structure with our tools.

There are four kinds of interventions introduced in this article, the low molecular weight heparin (LMWH), the Apixaban, the Rivaroxaban and the Edoxaban. All these four interventions could be used for treatment of cancer associated venous thromboembolism. The paper shows the expectation of costs and QALYs (12 months) of all these four interventions, and we can roughly extract the coefficient of variation (CV, standard error divided by mean) form the confidence intervals and simulation figures. With some more assumptions, like $\lambda = 30000$, the parameters in our example are shown in Table 2.

**Table 2:** Parameters for empirical example

	Cost	Var_of_Cost	QALY	Var_of_QALY	$\rho_{QC}$			
LMWH	21,512.00	41,648,952.96	0.53	28.09	0.1			
Apixaban	1,944.00	340,122.24	0.55	30.25	0.1			
Rivaroxaban	2,122.00	405,259.56	0.53	28.09	0.1			
Edoxaban	1,968.00	348,572.16	0.52	27.04	0.1			

	$\rho_C$				$\rho_Q$			
	LMWH	Apixaban	Rivaroxaban	Edoxaban	LMWH	Apixaban	Rivaroxaban	Edoxaban
LMWH	1	0.005	0.005	0.005	1	0.005	0.005	0.005
Apixaban	0.005	1	0.02	0.02	0.005	1	0.02	0.02
Rivaroxaban	0.005	0.02	1	0.02	0.005	0.02	1	0.02
Edoxaban	0.005	0.02	0.02	1	0.005	0.02	0.02	1

To show the benefit from mixed interventions and the optimality of our proposed structure, we compare six situations. Four of them are using these four interventions alone, the fifth is an equal weighting structure and the final is the structure generated from our tool. The mean, variance and the return-to-risk ratio are calculated for all these six situations, and the results are shown in Table 3.

**Table 3:** Results for empirical example

	Population benefit (mean)	Population benefit (variance)	Return-to-risk
LMWH only	− 5612.00	2.55E+10	− 0.035
Apixaban only	14,556.00	2.72E+10	0.088
Rivaroxaban only	13,778.00	2.53E+10	0.087
Edoxaban only	13,632.00	2.44E+10	0.087
Equal weight mix	9088.50	6.64E+09	0.112
Optimal structure	13,981.95	8.87E+09	0.148

We can find in Table 3 that none of the four interventions alone could make the return-to-risk ratio of population benefit more than 0.100, but just a simple mix with equal weight can make it 0.112, which clearly shows the benefit from mixing interventions. Moreover, simple mix does benefit, but only the optimal structure generated by our tool reaches the highest ratio of 0.148. This is around 0.7 times higher than solely use the intervention suggested by classical CEA tools.

The optimal structure is {0.00%, 32.62%, 33.22%, 34.16%}, which gives zero weight on LMWH and nearly equal weights on Apixaban, Rivaroxaban and Edoxaban. This consistent with the judgment of the original article that the intervention of LMWH is dominated. This also shows the reliably of our tool.

## Discussion

The tool proposed in this paper may suggest the population to use mixed interventions instead of a unique one. The population benefit can result in a controlled risk level due to diversification effect. To suggest mixed interventions in the real world has many considerable benefits, and still has kinds of challenges.

Besides risk consideration, mixed interventions could give the population a chance to accept an innovated intervention gradually, to avoid potential long-tailed losses, such as some long-term adverse effects that have not yet been discovered by clinical trials (such as the example of Thalidomide). Moreover, unique intervention decisions could result in high concentration risk and the potential monopolies. An intervention provider who won the winner-takes-all game can make a much higher price without competitors. Third, there is also heterogeneity in patients' willingness to pay and mixed interventions leaves room for patients to make their own decisions. At last, mixed interventions tend to give chance to innovated interventions, even just a little part of the market, to encourage technological advances.

However, it is necessary to face up to the challenges of mixed interventions. First, mixed interventions may confuse clinical decision makers in making a finally decision for individuals. This challenge could be settled by give soft suggestions with room for choice. Clinical decision makers could select the interventions accepted in the mixed interventions with their own expertise. In-depth differential diagnosis and intervention may yield better outcomes. Second, mixed interventions may force the healthcare settings to prepare the ability to provide all kinds of interventions in the mix, which will cause a lot of waste in resources. This can be solved by an integrated management. We can suggest a big hospital A to provide robotic surgeries and allow a primary care setting B to provide minimally invasive ones. Interventions could be allocated according to their own preference and specialties. The mix should be kept in an integrated view. Third, it can be difficult to keep the structure of mixed interventions at the optimal point. That is also a reason that we proposed a marginal adjustment tool. The real world is dynamic, and we should manage it dynamically. Even we can’t always keep an optimal structure, we can make slight adjustment according to the marginal adjustment criterion proposed in this paper to get a better population benefit.

Also, some statistical tools might be introduced in real world estimation of the parameters used in our method. For the correlation between costs and outcomes for individuals, it is already contained in classical HTA data records, but just neglected in classical assessment processes. For correlation among different patients, retrospective statistical analysis in the real world can help to get the estimations. Moreover, sensitivity tests can examine the reliably of results. More econometric tools can help in estimating systematic uncertainties.

At last, it is worth to mention that the tool we proposed has a good property of generalization. Although the criterion is named as risk-adjusted expected costs, we consider all kinds of risks and uncertainties for interventions including but not limited to costs and outcomes. Moreover, this tool support for all types of risk measures, which means that the decision could be made in any level of risk aversion. Also, the criterion, risk-adjusted ICER, retains the same expression form as the classical ICER, keeping high comparability with previous conclusions and low tool substitution costs. The risk-adjusted expected cost used in the numerator also has clear economic implications. The risk adjustment item added to the expected cost is obtained by multiplying the cost rate of risk by the allocated risk level, which is consistent with the economic logic of risk premium. And finally, it is not sensitive to the measure of life quality and currency.

## Conclusion

This paper proposes a stochastic CEA tool that considers uncertainty from the perspective of population benefits. The tool can evaluate the cost-effectiveness of different interventions under the current intervention structure, considering all kinds of risks in costs and outcomes. In theory, we present a necessary conditions for achieving the optimal intervention structure that maximizes the return-to-risk ratio of population benefits. Furthermore, we introduce the risk-adjusted ICER as a criterion for comparing any two interventions under any structure of mixed interventions. Theoretical analysis confirms that the criterion is optimizing population benefits. Results from numerical simulations and empirical examples also support these findings.

The population benefit gives a new perspective on measuring the cost-efficiency of a population but not individuals. And the tool we proposed helps to find the optimal mixed structure of interventions to maximize the return-to-risk ratio of the population benefit. The risk-adjusted ICER as the criterion can effectively induce the optimization and has clear economic implications. The conclusions obtained by the method proposed in this paper in the CEA analysis with uncertainty should be provided as decision reference to medical decision-makers. Acknowledgements: Kindly check and confirm whether the “Preprint Acknowledgements” statement is correctly processed.Yes, the address of the preprint and the statement is correctly processed.

## Data Availability

Not applicable.

## Abbreviations

CEA: Cost-effectiveness analysis
ICER: Incremental cost-effectiveness ratio
CEAC: Cost-effectiveness acceptability curve
NMB: Net monetary benefit
RAPM: Risk-adjusted performance measurement
CERAC: Cost‑effectiveness risk-aversion curve
QALY: Quality-adjusted life-year
VaR: Value at risk
CTE: Conditional tail expectation

## Footnotes

1 The bias in the comparison results of this pair of interventions is a random result with a small probability due to the limited number of samples.
